# Linking brain activity across scales with simultaneous opto- and electrophysiology

**DOI:** 10.1117/1.NPh.11.3.033403

**Published:** 2023-09-01

**Authors:** Christopher M. Lewis, Adrian Hoffmann, Fritjof Helmchen

**Affiliations:** a University of Zurich, Brain Research Institute, Zurich, Switzerland; b University of Zurich, Neuroscience Center Zurich, Zurich, Switzerland; c University of Zurich, University Research Priority Program, Adaptive Brain Circuits in Development and Learning, Zurich, Switzerland

**Keywords:** multimodal, multiscale, electrophysiology, optical imaging

## Abstract

The brain enables adaptive behavior via the dynamic coordination of diverse neuronal signals across spatial and temporal scales: from fast action potential patterns in microcircuits to slower patterns of distributed activity in brain-wide networks. Understanding principles of multiscale dynamics requires simultaneous monitoring of signals in multiple, distributed network nodes. Combining optical and electrical recordings of brain activity is promising for collecting data across multiple scales and can reveal aspects of coordinated dynamics invisible to standard, single-modality approaches. We review recent progress in combining opto- and electrophysiology, focusing on mouse studies that shed new light on the function of single neurons by embedding their activity in the context of brain-wide activity patterns. Optical and electrical readouts can be tailored to desired scales to tackle specific questions. For example, fast dynamics in single cells or local populations recorded with multi-electrode arrays can be related to simultaneously acquired optical signals that report activity in specified subpopulations of neurons, in non-neuronal cells, or in neuromodulatory pathways. Conversely, two-photon imaging can be used to densely monitor activity in local circuits while sampling electrical activity in distant brain areas at the same time. The refinement of combined approaches will continue to reveal previously inaccessible and under-appreciated aspects of coordinated brain activity.

## 1 Introduction

**Fig. 1:**
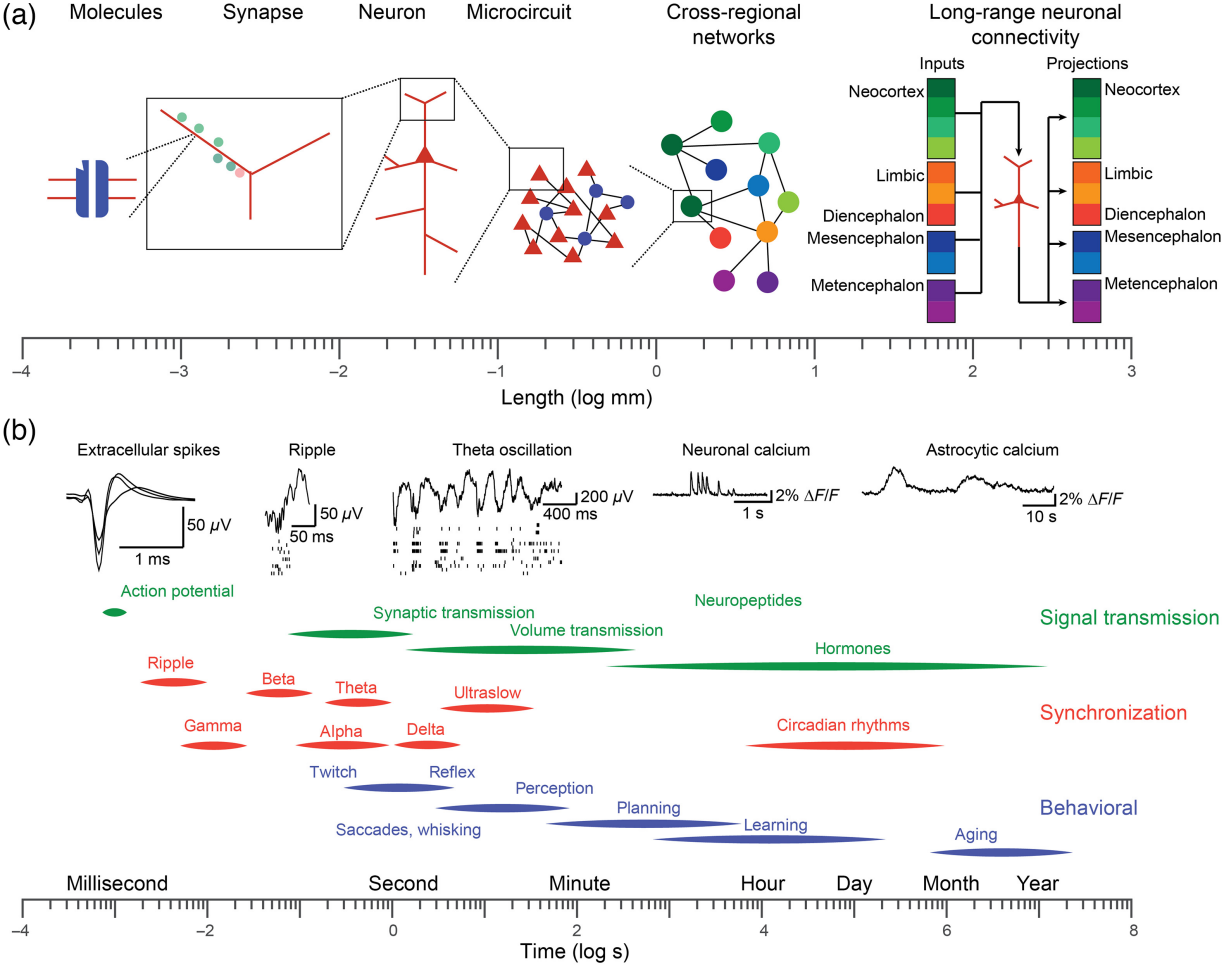
The brain has precise organization across spatial and temporal scales. (a) The brain spans many orders of magnitude in spatial organization, from subcellular features, such as ion channels and dendritic spines, to single neurons, local networks of heterogeneous neurons and non-neuronal cells, such as glia, to long-range synaptic and non-synaptic projections between highly distributed brain regions. Single neurons within a local microcircuit may receive input from and transmit signals to an idiosyncratic constellation of local and long-range pre- and post-synaptic neurons. (b) Likewise, brain activity and behavior are organized across a wide range of temporal scales from individual spikes and synaptic events in the millisecond regime, to synchronization of populations at distinct temporal scales, all the way to perception, decision making, and learning, which can occur across minutes, days, and years. Unpublished data from C. Lewis.

Biological systems are astronomically complex. They are composed of a profusion of diverse cells that are organized with exquisite precision to transform, store, and communicate information using a rich repertoire of signals. This complexity is perhaps nowhere more evident than in the brain, with nearly as many neurons in each human brain as there are stars in the Milky Way galaxy ($∼10^{11}$). The interactions between cells within local circuits and between distributed populations give rise to intricate dynamics across a wide range of spatial and temporal scales (Fig. 1).^1^^–^^7^ Single neurons are highly diverse, varying both genetically and transcriptionally, as well as in their morphology and electrophysiology.^8^^–^^11^ Neurons are organized and connected in a highly specific manner^12^^–^^15^ and interact not only with each other—over short and long distances—but also with non-neuronal cells in their vicinity, such as astrocytes and microglia.^16^^–^^18^ Individual neurons also have unique patterns of local and long-range synaptic inputs and outputs,^8^^,^^12^^–^^15^ which determine the information they receive, the computations they perform, and the signals they transmit to downstream cells [Fig. 1(a), right]. These synaptic and non-synaptic influences combine with the electrophysiological characteristics of single cells to give rise to diverse activity patterns at a variety of time scales from milliseconds up to tens of seconds [Fig. 1(b)]. Likewise, the behavior of organisms is structured in time from rapid muscle twitches and fixational eye movements to extended periods of perception, planning, learning, and aging that occur across minutes, hours, days, and years. To understand the role of single neurons in the brain-wide activity patterns that underlie adaptive behavior across these spatial and temporal scales, it is desirable to track the activity of single neurons, as well as population activity from distributed brain regions across days and months.

Historically, systems neuroscience has focused on recording the activity of single neurons or bulk activity from single regions of interest.^19^ This limitation was largely due to the lack of methods to record large numbers of cells or to monitor non-electrical activity in the brain with high spatial resolution. However, it was also motivated by the belief that brain activity could be understood in a reductionist manner, in terms of the tuning of single cells,^20^^,^^21^ organized in generic circuits,^22^ and within domain-specific regions.^23^^,^^24^ This belief was exemplified by the search for single-neuron correlates of perception, domain-agnostic canonical circuits, and an emphasis on functional localization. While these studies have taught us a great deal about the properties of single cells, the organization of local circuits, and the more global organization of the brain, there has been an increasing appreciation for how the diversity and specificity of cells and circuits give rise to rich, highly dynamic activity patterns across widely distributed brain networks.^25^^–^^27^ A variety of new tools have expanded experimental access to monitor and perturb activity in the intact brains of behaving animals over the past years.^14^^,^^26^^,^^28^^–^^36^ New methods to record spike patterns in large populations of neurons, to monitor diverse cellular signals, and to map structural connectivity at unprecedented scales have revealed the intricate, heterogeneous organization of brain circuits.^12^^,^^13^^,^^27^ New anatomical data have emphasized that most neurons, while preferentially connected with nearby cells, also receive and send extensive and idiosyncratic long-range connections.^8^^,^^37^^–^^40^ For example, neighboring neurons in the primary somatosensory whisker cortex of the mouse can have distinct patterns of connectivity, and single thalamic neurons can project to highly distributed constellations of brain areas.^14^^,^^41^^,^^42^ Further, even in early sensory areas, the response properties of neurons are not static but undergo prominent context- and state-dependent changes that might arise from long-distance neuronal and neuromodulatory projections, or through the effects of local non-neuronal cells, such as astrocytes.^25^^,^^43^^–^^49^ These studies have increased our appreciation for the diversity and specificity of neuronal connectivity, as well as the precise coordination of activity in populations of cells. However, most studies are limited to monitoring a single signal of interest in one or a few brain areas. Understanding how the activity of single neurons is embedded within the distributed activity patterns of whole-brain networks and how dynamics on these whole-brain networks enables robust, yet adaptive behavior requires the integration of multiple measurement modalities, both to monitor the diverse molecular signals used by the brain and to bridge different scales of organization.

In this review, we first highlight the strengths of different measurement techniques and how the strengths and weaknesses of individual techniques can be combined to complement each other. Based on this complementarity, we then motivate the combination of electrophysiological and optical measurements of brain activity toward understanding integrated brain function. We specifically discuss recent advances in the fabrication of multi-electrode arrays (MAEs) that make them more conducive to combination with optical methods, also considering the challenges to keep in mind when planning a new multimodal experiment. We then outline recent studies, from the mouse brain, that have leveraged the combination of electrical and optical recording of brain activity to reveal new principles of brain function. Finally, we discuss how multimodal data can be analyzed to provide new insights and emphasize the need for new analysis and modeling approaches that permit multiscale analysis of brain data.

## 2 Motivation

### 2.1 Different Measurement Modalities Have Complementary Strengths and Limitations

All measurement techniques have trade-offs and relative strengths and weaknesses. These factors depend on the features of the measurement apparatus, such as its spatial and temporal resolution, the accessible coverage [field-of-view (FOV) for imaging methods], and the maximum tissue depth reachable by the respective technology. However, the limitations of a given measurement also depend on the spatial and temporal characteristics of the specific physiological activity measured, as well as on how the activity is transduced into a measurable signal. Both electrical and optical techniques can assess signals across a broad range of temporal and spatial scales depending on their precise technological implementation and application (an overview is presented in Table 1). Here, we review the main electrical and optical recording methodologies and their respective strengths and limitations, as well as how these distinct strengths can be complementarily combined. Finally, we motivate multimodal investigations of brain function by highlighting two areas in which multimodal experiments have been crucial to yield new perspectives on brain function.

### 2.2 Strengths and Limitations of Electrical Measurements

**Fig. 2:**
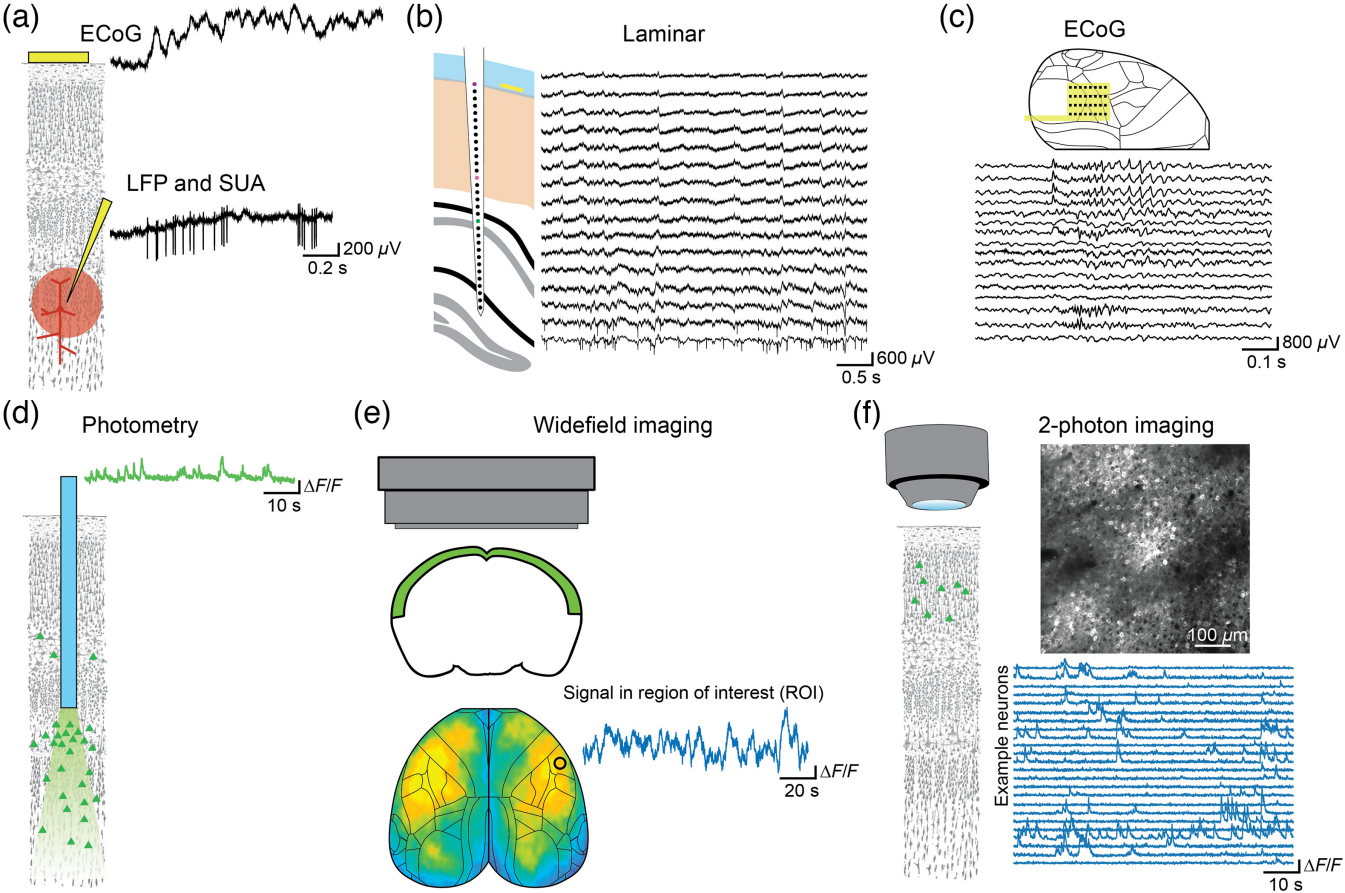
Diverse electrical and optical recording methods. (a) Top: electrical recording of mesoscopic, lowfrequency activity from the surface of the brain (ECoG). Bottom: recording of lowfrequency (LFP) and high-frequency (MUA/SUA) from an intracortical electrode. (b) Multi-electrode recording of depth-resolved, laminar electrical activity from a linear electrode array. (c) Multi-electrode recording of the electrical activity across the surface of the brain with an ECoG array permits topographical investigation of neuronal activity tangential to the depth. (d) Fiber photometry records the bulk fluorescence of a genetically encoded activity indicator from a population of cells around the tip of the fiber. (e) Widefield imaging records the mesoscale, population activity of a genetically encoded activity indicator across the dorsal cortex. (f) Two-photon imaging can resolve cellular and subcellular activity from populations of identified single cells expressing a genetically encoded activity indicator. Unpublished data from C. Lewis and A. Hoffmann.

Electrophysiological recordings of neuronal activity have been the gold standard for systems and circuit neuroscience over many decades.^50^^–^^52^ They offer excellent temporal resolution (microseconds), and the spatial scale of the measurement can be varied across a broad range, from single ion channels to macroscopic measurements of electrical signals in neuronal populations [Figs. 2(a)–2(c)]. In addition to measurements, electrodes can be used to stimulate activity in the local population (or even of a single neuron) around the electrode tip, although care should be taken to ensure that stimulation does not lead to deterioration of the electrode or damage the tissue. The highest fidelity recordings are achieved with intracellular recording electrodes that can measure both the supra- and subthreshold activity of single neurons and permit the determination of morphology and genetic information. However, the use of intracellular electrodes to measure *in vivo* brain activity is currently limited to the measurement of single cells and is difficult to combine with chronic imaging in behaving animals over long time periods (but see Ref. 53; modified cranial windows make it feasible^54^). For the purposes of this review, we focus on extracellular recordings with MEAs. MEAs are limited to the measurement of voltage differences outside of neurons with a spatial resolution that depends on the size and density of the recording contacts. A wide variety of arrays are commercially available, with the majority fabricated using lithography to structure conductive materials on rigid silicon, although flexible arrays based on polymers, such as polyimide or silicones, are increasingly common.^34^^,^^55^^,^^56^ MEAs can record a wide range of electrical signals that roughly correspond to different spatial scales, from the action potentials (APs) of single, isolated units (single-unit activity, SUA), to the aggregate APs of small populations of cells (multi-unit activity, MUA) [Figs. 2(a)–2(c)].^57^ The extracellularly recorded signal is commonly split into at least two frequency ranges, with the activity above ∼500  Hz considered to reflect spikes (which still need to be sorted^58^^,^^59^) and that below ∼300  Hz referred to as the local field potential (LFP), which is considered to reflect predominantly subthreshold (dendritic) activity. The number of isolated, single neurons that can be recorded depends primarily on careful implantation, as well as on the number and spatial arrangement of the electrode contacts on the array.^59^^,^^60^ With single-wire electrodes, the typical yield is one to three isolated units; however, dense arrays are better able to distinguish the activity of intermingled neurons. The neuron yield of modern MEAs, with hundreds of electrode contacts, has increased to hundreds or even thousands of units.^52^^,^^59^^–^^61^ In addition, the LFP can be further subdivided into frequency bands of interest, which reflect the synchronization of populations of neurons at distinct time-scales and can help define oscillatory states.^2^ Linear MEAs can provide additional spatial information about the distribution of neuronal activity across the depth of the cortex [Fig. 2(b)]. Intracortical arrays can be acutely inserted into the brain during an experiment, or they can be chronically implanted. In general, acute placement permits flexibility to probe different populations across sessions and does not require the fixation of bulky interconnects onto the skull, which permits the placement of more arrays simultaneously (typically one to three, sometimes up to six^62^^,^^63^). However, chronic implantation permits longitudinal measurement of neuronal and population activity and easy connection to recording equipment without the need for repeated craniotomies and probe placement. The size of the skull defines the available space for craniotomies and probe placement and limits the number of locations that can be simultaneously monitored with MEAs, especially for chronic implants. Recordings using rigid MEAs are typically limited to a single chronic penetration, whereas flexible arrays can be adapted to target multiple targets.^64^ MEAs placed on the surface of the brain or in contact with the dura mater can record the electrical potential changes of superficial brain structures; a local variant of electro-encephalography (EEG) commonly referred to as electro-corticography (ECoG) [Figs. 2(a) and 2(c)]. The ECoG signal predominantly consists of low-frequency (LFP) activity but depending on the electrode size and array-brain coupling, ECoG arrays placed on the surface of the brain can also record APs from superficial neurons^65^ and can be adapted to cover large portions of the dorsal cortex.^66^^,^^67^ While most arrays are passive, meaning that each contact is coupled to a wire that conveys the signal out of the brain to the recording system, recent advances, such as the Neuropixels or Neuroseeker probes, have greatly increased the number of recording contacts by introducing temporal multiplexing to produce arrays of unprecedented density.^36^^,^^68^

Despite their variety and strengths, electrophysiological measurements have some notable drawbacks. First and foremost, they can only record voltages (voltage differences in the case of extracellular electrodes, whereas absolute voltages and ionic currents can be recorded with intracellular electrodes) so the activity of non-spiking or electrically silent cells cannot be monitored. Likewise, it is difficult to monitor neuromodulatory activity, although electrochemical methods such as fast cyclic voltammetry provide some access using specialized electrodes.^69^ In addition, an extracellular electrode records the sum of all the electrical activity in its vicinity (from a volume that depends on its size and electrical characteristics) and is blind to the source of the signals it records. It is therefore difficult to determine the identities of the recorded cells, although it is possible to broadly discriminate between cells based on the AP waveform, firing rate, or, in the case of dense recordings, the inhibitory or excitatory effect they have on other simultaneously recorded cells.^70^^,^^71^ The highest degree of specificity is achieved by electrical recordings of isolated cells that express an opsin, enabling them to be identified based on their response to optical stimulation (a procedure known as “opto-tagging”).^72^^–^^74^ In general, electrical recordings are also invasive, as a foreign object must be placed in contact with or inserted into the brain (or in proximity as for EEG electrodes). For inserted electrodes, which have the best resolution and are generally required to detect APs, the mechanical disturbance and the foreign material may create acute damage to the cells and neuronal processes in its vicinity.^75^^,^^76^ Chronically, inflammation of the local tissue may be induced by the presence of the foreign body, especially when there is a mechanical mismatch between the elasticity of the tissue and the rigidity of the electrode material. For chronic implantations, a permanent route from the outside of the skull to the inside of the brain provides a potential path for pathogens and care must be taken to establish a sterile and robust interface that remains stable for the duration of the experiment. These factors also affect the ability to perform long-term recordings of isolated single units across days and months with intracortical MEAs. While it is possible to perform long-term recordings, their stability depends on both the reaction of the tissue to the implantation, as well as the movement of the brain with respect to the array over the life of the implant. Increasing the density of arrays can permit the tracking of isolated units that move in relation to the rigid array,^60^ whereas the use of flexible arrays may improve the long-term electrode–tissue interface and reduce motion of the array relative to the brain.^64^^,^^77^ Despite these limitations and drawbacks, extracellular recording of neuronal activity with high-density MEAs provides the highest temporal resolution, flexible targeting of deep and superficial brain areas and remains an essential recording method for understanding local and distributed neuronal activity.

### 2.3 Strengths and Limitations of Optical Measurements

In parallel to the advancements in electrophysiology, the past decades have seen rapid improvements and increased application of optical methods to monitor and manipulate brain activity. Optical recordings have several distinct advantages to electrical recordings. First, they can measure a wide range of physiological signals depending on the specific contrast agent. Optical methods can leverage intrinsic contrasts, for example, the wavelength-dependent absorption or reflectance of hemoglobin to assess hemodynamics and autofluorescence, or secondharmonic or third-harmonic generation to image anatomical features.^78^ Most commonly, fluorescent indicators are used to indirectly measure a physiological signal of interest, such as voltage, or concentration changes of signaling ions or molecules, such as calcium, chloride, protons (pH), glutamate, acetylcholine, dopamine, serotonin, Adenosine triphosphate (ATP), camp cyclic adenosine monophosphate (cAMP), and many more.^79^ Classically, suitable organic or inorganic dyes were identified and tailored to sense voltage and calcium, but the identification and modification of fluorescent proteins have given rise to an increasing range of diverse genetically encoded sensors with increased sensitivity, brightness, and specificity.^80^ Most notably, the continued development of the GCaMP family of indicators has revolutionized the recording of large populations of neurons.^81^^,^^82^ Of interest for population recording is the reliable estimation of neuronal firing rates based on calcium-dependent fluorescence changes,^83^ as such extensive effort has gone toward the iterative improvement of genetically encoded calcium indicators to increase their signal-to-noise ratio and increase their temporal precision. New methods for high-throughput screening, directed evolution of sensors, and protein engineering continue to drive the development of new fluorescent sensors.^84^^,^^85^ Together with viral techniques for transgene expression and transgenic technology, the use of genetically encoded sensors provides a powerful means to specify the expression of indicators in identified cell types, including specific projection pathways or even specific subcellular domains.^86^ This specificity is a key benefit of optical methods and has been used to identify new classes of cells and to begin to uncover cell-type specific functions. An additional advantage of optophysiology is that the wavelength-dependence of optical sensors permits spectral multiplexing. Because each sensor has characteristic excitation and emission spectra, in principle, multiple sensors can be used to record diverse physiological signals simultaneously from the same volume. However, in practice, care must be taken to ensure minimal crosstalk between independent fluorescent reporters, and sensor performance may not be homogeneous across the spectrum.^85^

Optical techniques can be broadly divided based on whether they form an image of the sampled tissue or not. Non-image-forming techniques, such as photometry, record the bulk fluorescence signal by exciting and capturing light emission through a single optical fiber, producing a single time series [Fig. 2(d)]. The insertion of the optical fiber into the brain, which is required to target subcortical regions, may have a similar impact on the tissue as the implantation of electrode arrays. By using multiple fibers in parallel,^87^^–^^89^ several locations can be measured at the same time. Image-forming methods produce a spatial map of activity, either by forming an image directly onto a two-dimensional (2D) sensor, capturing image stacks or movies, or by creating a 2D image (or 3D volume) through sequential scanning of a focused laser beam^90^^,^^91^ [Figs. 2(e) and 2(f)]. Image-forming methods are therefore beneficial for spatially resolving the signal of interest but vary widely in their resolution, addressable volume, and coverage. Widefield imaging enables broad imaging from large fields-of-view with sub-second temporal resolution and has been increasingly applied to image population activity across the dorsal cortex of mice [Fig. 2(e)].^90^ The frame rate of widefield imaging is defined by the camera, and state-of-the-art cameras can achieve rates of up to a few hundred Hz when imaging a subset of the pixels. However, very high frame rates are primarily important when imaging fast indicators, for example, genetically encoded voltage indicators (GEVIs),^92^^,^^93^ and frame rates in the range of tens of Hz are standard and sufficient when imaging bulk fluorescence signals from neuronal populations expressing calcium indicators, which typically have slower kinetics (≥100  ms decay time constants). Often widefield imaging lacks cellular resolution, with the signal in each pixel representing the summed activity of many cells. Special preparations can enable imaging from smaller areas with higher spatial resolution.^94^^,^^95^ On the other hand, two-photon imaging increases spatial resolution at the cost of smaller fields-of-view and more complicated imaging systems [Fig. 2(f)]. Two-photon imaging systems have experienced rapid development over the past decades, and in combination with transgenic mouse strains and high-performance indicators, it is possible to record thousands of cells in a session with reasonable frame rates (ten to twenty Hz with standard resonant-galvanometer scanning, but up to kilohertz with more complicated scanning systems^96^^,^^97^). The combination of better indicators and the wide availability of microscopes (either custom-built or suitable commercial ones) have made two-photon microscopy a go-to method for recording large populations. In general, imaging provides additional information about topographic organization (functional maps) or the morphology of imaged cells. Critically, cellular imaging provides the ability to track the same cells or even subcellular compartments longitudinally across days and weeks,^98^^–^^101^ permitting the evaluation of cellular and population dynamics over extended periods. While care must be taken to align the imaging FOV across sessions, there are tools to perform flexible alignment of cellular imaging datasets acquired across sessions.^102^^,^^103^ Further, image-forming optical methods present an opportunity to perform post-hoc anatomical, histological, or *in-situ* sequencing analysis of physiologically characterized cells, providing an additional dimension and degree of specificity.^104^^–^^106^ The spatial and temporal resolution, FOV, and tissue depth penetration of imaging-forming techniques depend on the specifics of their optical system (especially on magnification and the numerical aperture, NA), scan technology (line-, raster-, or random-access scanning), and on whether single-photon or multiphoton excitation is used. All image-forming techniques have tradeoffs between the sampling rate of the system (how fast images are acquired), the FOV that can be covered (the area or volume sampled), and the signal-to-noise ratio, which depends on fluorophore characteristics and on how much time is spent on sampling photons from a given location.^107^

Most image-forming techniques are limited to the surface of the brain due to scattering and absorption of light in tissue. Even two-photon microscopy barely reaches 1-mm imaging depth^108^^–^^111^ but advanced techniques, such as three-photon microscopy^109^^,^^112^^,^^113^ and photoacoustic imaging^114^^,^^115^ (with lower resolution), are pushing the limit for deep imaging. Alternatively, imaging techniques can be combined with the removal of superficial areas and/or the chronic implantation of cannulae or gradient-index (GRIN) lenses to provide spatially resolved optical access to deep structures. Such approaches are powerful, but the removal of the overlying brain structures increases the invasiveness of the approach and can affect the behavior of the animal depending on which structures are affected. The past decade has seen the rapid development of new imaging methods to record from large populations of neurons. This trend is apparent in the increased application of widefield imaging and in the development of new volumetric single-cell imaging approaches, based on temporal focusing, multifocal and multiplexed scanning, plane-hopping, or the use of special objectives for imaging larger FOVs.^116^^–^^119^ Advances in the imaging of single cells and cell populations across ever-increasing volumes provide a fertile ground for combination with recent developments in fabrication of MEAs.

### 2.4 Why Perform Multimodal Studies?

We have highlighted the unique strengths and limitations of the diverse electrical and optical recording methods commonly used in systems neuroscience. We believe that the distinct strengths and challenges of electrical and optical recording modalities complement each other (see Table 1 for an overview) and provide abundant opportunities for synergistic application. As noted, electrical recordings provide excellent temporal precision, including single AP resolution, and access to population activity, such as sub-second synchronization within and between neuronal populations. In addition, MEAs provide robust access to deep brain structures, which are difficult to measure optically, requiring relatively invasive approaches. In contrast, optical methods can provide genetic targeting of identified cell-types, viral-based targeting of identified projection pathways, and unbiased, high-throughput recording of single cells, even with sub-cellular spatial resolution. Alternatively, imaging can sacrifice resolution in favor of broad imaging of cortex-wide dynamics. The possible combinations are wide-ranging, either zooming in to monitor distinct signals within a local population or zooming out to monitor the flow of activity across whole-brain networks. For example, within a local population, one could investigate how a single cell type or projection axon participates in the mesoscopic synchronization reflected in oscillatory patterns of the LFP. Alternatively, zooming out, one could simultaneously monitor the activity of distinct cortical and subcortical nodes of a brain-wide network, for example, the coordinated activity across a sensory or motor hierarchy (for more examples of key open questions, see Box 1). Before highlighting specific recent examples of combined electrical and optical investigation of brain function, we illustrate two domains in which pioneering multimodal studies have contributed to our understanding of basic brain function.

Box 1. Key open questions

• How do idiosyncratic local neurons give rise to coherent patterns of activity?
• How do single neurons integrate diverse inputs arising from distributed presynaptic partners?
• What information do individual neurons contribute to brain-wide activity patterns?
• How are distributed neurons organized into large-scale brain-wide states?
• Do computations and transformations take place in local circuits, or do large-scale distributed networks perform computations?
• How are brain-wide states dynamically regulated and what determines the transition between states?

### 2.5 Two Domains Revolutionized by Multimodal Studies

Pioneering multimodal experiments, many of them combining electrophysiological and optical measurements, have already contributed significantly to our understanding of brain function. They have been especially useful in addressing two fundamental questions: (1) How is the brain’s ongoing, intrinsic activity organized and how does it affect neuronal responses? (2) How does neuronal activity relate to hemodynamics via neurovascular coupling? In a series of seminal experiments, Grinvald, Arieli, and colleagues^120^^,^^121^ combined electrical recordings of single neurons in the primary visual cortex with voltage-sensitive dye imaging of the cortical surface. These studies indicated that the considerable firing rate variability of sensory neurons to identical stimuli does not reflect “noise,” as had been classically thought, but rather is highly structured in space and time and largely predictable based on the recent history of activity in the local population. These insights fundamentally challenged the dominant conceptual framework in sensory neuroscience, motivating a move away from the stimulus-response logic of most experiments and toward a perspective, in which the brain’s ongoing dynamics influence the individual neuron’s response to sensory stimulation.^122^^–^^124^ Another set of studies combined intrinsic optical imaging of hemodynamic activity in the brain with electrical recording of single neurons or neuronal populations to better understand the basis of neurovascular coupling. These studies revealed that hemodynamic signals are nonlinearly coupled to neuronal activity, often more closely associated to features of the LFP,^125^^,^^126^ which might reflect dendritic activity,^127^ or activity in adjacent populations,^128^ rather than simply reflecting the firing rates of neurons in the local population.^129^^–^^131^ In addition, the activity of glial cells plays a key role in neurovascular coupling and has been associated with distinct components of the hemodynamic signal.^132^^–^^135^ Most striking are instances in which neuronal activity is decoupled from local blood flow, suggesting complex, highly dynamic coupling that can vary based on sensory and cognitive context.^125^^,^^127^^,^^128^^,^^132^^,^^136^^–^^140^ Indeed, rather than solely representing a spatially and temporally blurred proxy for neuronal activity, hemodynamic signals may be linked to other aspects of brain function by responding to and influencing neuromodulatory signals (neurotransmitters, neuropeptides, ${\mathrm{NO}}_{2}$) and non-neuronal cells, such as glia.^127^^,^^132^^,^^141^ Solving these still-open questions concerning the complex interplay between neuronal and vascular activity will require the continued application of multimodal studies. While new all-optical approaches^142^ and the combination of electrophysiology or optical methods with functional MRI (fMRI)^143^^,^^144^ can add important insights to this and other topics, we believe that the combination of electrical and optical measurements will continue to play an important role because of their previously noted complementary strengths, specifically, the ability of optical methods to monitor a diverse range of cellular and subcellular signals and processes and the unparalleled temporal resolution of electrical measurements.

In addition to these two areas, simultaneous electrical and optical measurements promise to contribute to our understanding of a wide range of urgent neuroscience questions. Recent work has begun to use combined measurements to reveal how the activity of single neurons is coordinated with brain-wide activity patterns, how brain activity is organized across spatial and temporal scales, and how inputs are transformed in local circuits and transmitted to distributed partner regions to enable coordinated whole-brain states.^145^^–^^148^ Here, we propose that the further development and application of multimodal techniques will make these questions about neural computation more amenable to experimental analysis and thereby promote scientific insights into the organizing principles of integrated brain activity. We expect insights into the principles of hierarchical dynamics across local and distributed networks, including how the activity of disparate individual cells gives rise to coherent activity patterns, the regulation of brain state and behavioral states across the sleep-wake cycle, the ability, what the limits are of coarse-grained mean field estimates of brain activity, how single neurons integrate disparate input signals based on the state of the local circuit, and to what degree computations are implemented locally rather than distributed across brain-wide networks. The promise and utility of combined opto- and electrophysiology derives mainly from their complementary strengths and weaknesses. We next outline practical challenges for combined electrical and optical interrogation of brain function and highlight recent technical developments that may help circumvent some of these challenges.

## 3 Challenges of Multimodal Measurements and Recent Developments in Electrode Array Technology

**Fig. 3:**
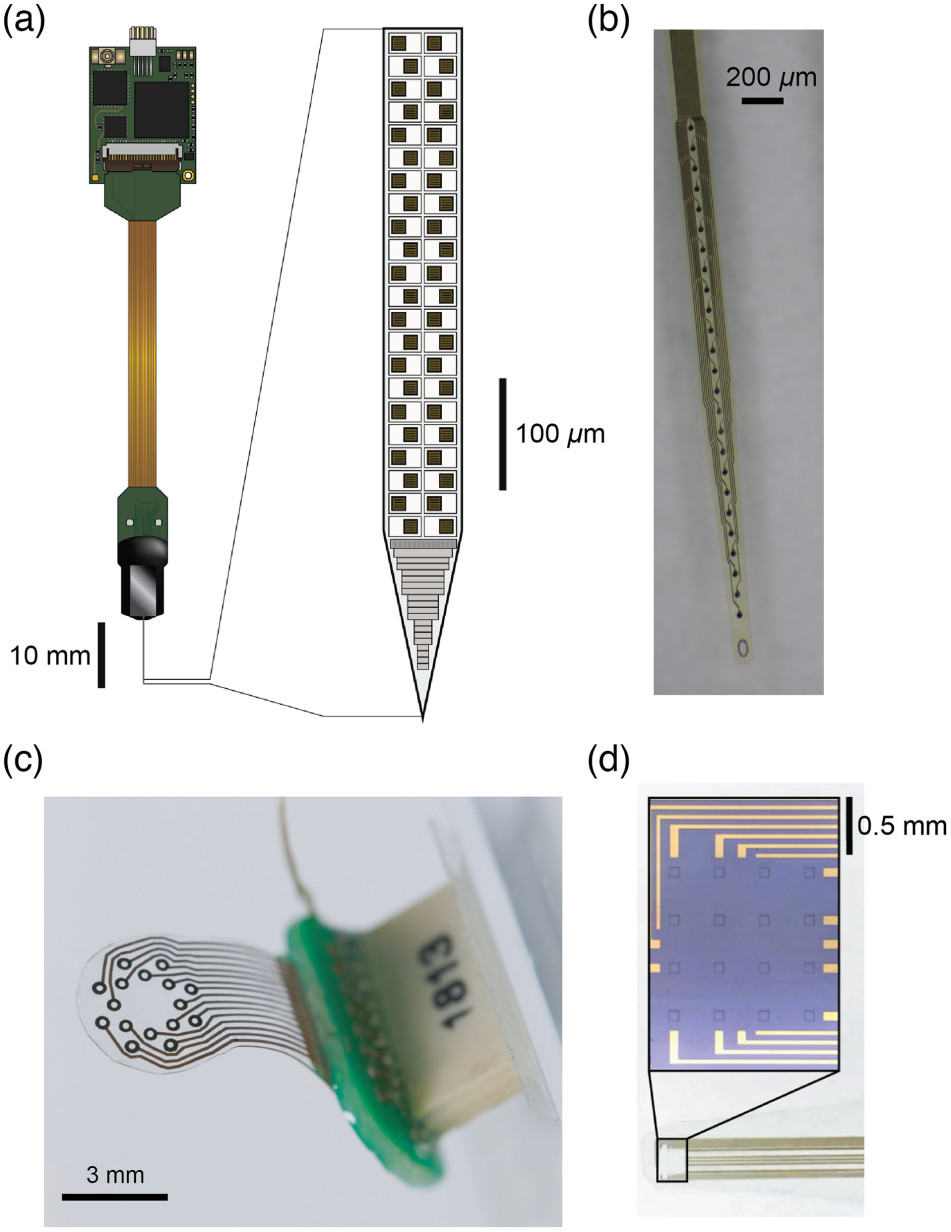
Novel MEAs enable combined electrical and optical recording. (a) High density MEAs, such as the Neuropixels array, permit recording from hundreds of electrodes across the length of the array. (b) Flexible and translucent or transparent arrays fabricated on polymers enable electrical recording from neurons in the depth of the brain, while still permitting the positioning of objectives in the probe vicinity for optical access to the local or distributed cells. (c) ECoG arrays fabricated on a stretchable and flexible transparent substrate permit recording of electrical activity from the surface of the brain, while enabling simultaneous optical access to the brain regions below the array. (d) ECoG arrays constructed using transparent conductors enable fully transparent electrode sites. Transparent electrodes permit optical access to the tissue directly below the recording site, enabling imaging from the cells giving rise to the mesoscale electrical activity recorded on the ECoG array. Electrode schematic and images adapted with permission from Refs. 36, 149, 67, and 150.

The combination of electrical and optical recording techniques in the same preparation requires careful consideration so the necessary apparati do not interfere with each other. Current and emerging advances in multi-electrode technology that render electrode arrays more conducive to the combination with optical methods make it a particularly exciting time to attempt multimodal investigations. New innovations in the fabrication of electrode arrays have improved the density of recording contacts, as well as introduced highly flexible arrays that match the mechanical characteristics of biological tissues or have substrates or electrode sites fabricated from transparent materials (Fig. 3). Each of these innovations offers different advantages that can complement optical recordings. Modern MEAs enable spatially and temporally resolved recordings with a precision down to tens of μm and <1  ms.^34^ Current high-density arrays, such as the Neuropixels probe, can contain hundreds of recording sites and enable high resolution recording of extracellular fields and spiking activity from hundreds to thousands of neurons [Fig. 3(a)].^36^^,^^63^^,^^68^ However, most commercial arrays are fabricated on a rigid silicon substrate that poses some challenges for the combination with optical recordings. The first challenge is mechanical, as the rigid geometry can constrain the simultaneous positioning of the array to an area of interest while maintaining optical access, e.g., with an objective lens. This constraint can be circumvented in situations where the objective lens has a large working distance (WD), enabling the array to exit the brain at a steeper angle, closer to the common vertical optical axis. While long-WD objectives often have optical limitations, improved objectives are being developed.^151^ In general, care must be taken that the light cone between objective and tissue is only minimally blocked by the array, connectors, or amplification and recording equipment (see Box 2, key challenges). Geometry is particularly a concern when chronic recordings are desired, as the probe and connector must be permanently adhered to the skull of the animal but should neither occlude the optical access nor constrain the animals’ freedom of movement when it is returned to its home cage. Another serious limitation of silicon MEAs is that most bulk silicon is sensitive to light. Direct light on a silicon array can lead to large voltage changes due to the photovoltaic effect.^152^ For some optical methods with constant illumination, such as widefield imaging, this is not a problem. However, for methods that require pulsed light with high peak powers (such as multiphoton imaging) and in situations in which the excitation light is flickered (either to increase sensitivity with a lock-in amplifier or to alternate wavelengths), illumination can lead to large artifactual transients superimposed on the physiological electrical recordings. In the case of multiphoton imaging with high laser intensities, these artifacts can saturate the dynamic range of the digitization and prohibit simultaneous measurement.

Box 2. Key challenges

• Geometric concerns of simultaneous MEA placement and high-NA water-dipping objective. (The placement of MEA connector while maintaining optical access is critical for chronic implants.)
• Potential light artifact depending on the MEA materials (can be mitigated for photometry, optogenetics, and widefield imaging by using low light intensities or altering the temporal profile to avoid transients; for two-photon imaging, requiring very brief laser pulses for maximum photon density, this is more difficult).
• Attention must be paid to the different spatial and temporal resolutions of electrically and optically recorded brain signals when performing multimodal analysis.
• There is a lack of practical and conceptual methods to mechanistically link diverse signals that reflect different levels of organization.

**Table 1:** Comparison of electrical and optical recording techniques.

	Intracortical MEA	ECoG	Photometry	Widefield	Two-photon
Temporal resolution	Sub-millisecond	Sub-millisecond	Sub-millisecond	Sub-second, typically in the range of 10 to 40 Hz	Sub-second, typically in the range of a few Hz to tens of Hz, special scanning systems exist to image up to 1 kHz
				Can use specialized cameras to record up to a few hundred Hz for GEVIs	
Spatial resolution	Records sum of activity within a sphere of a few hundred microns. Can distinguish single neurons from the shape of APs	Records sum of activity at the surface of the brain. In general, does not have cellular resolution. However, small electrodes can be used to record superficial neurons	Records the bulk fluorescence signal from a population around the fiber tip. Spatial scale is determined by the size of the fiber optic cannula and the expression of the indicator in the population	Can record activity from populations with a resolution determined by the imaging objective and scattering, but without optical sectioning	Sub-micron resolution in principle, can resolve single neurons and subcellular compartments, but practically depends on FOV
Longitudinal stability	Can record long-term from the same population, but unequivocal identification of the same neuron across time can be challenging	Can record long-term from the same population, but unequivocal identification of the same neuron across time can be challenging	Can record bulk activity from the same population for weeks	Can reliably image the same population for weeks	Can reliably image the same neurons and populations across days and weeks
Invasiveness	Mild, must perform a craniotomy and implant an array into the brain. Potential route for pathogens	Mild, must perform a craniotomy and implant an array over the surface of the brain. Potential route for pathogens	Mild, must implant a fiber optic cannula into the brain. Potential route for pathogens	Very mild, can image through intact skull	Mild, must implant a window for imaging cortex, or a cannula to image deep structures
Source separation	Single neurons can be distinguished, but not visualized. LFP is complex mixture of sources in volume around electrode	Complex mixture of sources in volume around electrode	Cannot distinguish individual components of bulk signal; however, expression of indicator can be targeted to a cell-type or projection	Cannot distinguish individual components of bulk signal; however, expression of indicator can be targeted to a cell-type or projection	Can visualize and segment cells and cellular processes from the neuropil. Expression can be targeted to specific cells
Flexibility of positioning	Can flexibly target brain regions of interest	Limited to the surface of the brain	Can flexibly target brain regions of interest	Limited to the surface of the brain or requires the removal of superficial tissue to access deep structures	Limited to 1 mm from the surface of the brain or requires the removal of superficial tissue to access deep structures
Damage to tissue	Can cause acute and chronic damage to tissue due to mechanical mismatch between array and brain	Can cause acute immune response upon implantation. Array can be encapsulated by dura-mater over time	Can cause acute and chronic damage to tissue due to mechanical mismatch between fiber optic cannula and brain	No or very little damage	Window can lead to acute immune response or surface bleeding, potential for tissue heating
Biases	Biased toward more active neurons if placing array acutely	Biased to superficial cells and mesoscopic potentials	Biased by viral tropism, indicator expression and distribution	Biased by viral tropism, indicator expression, and distribution	Biased by viral tropism, indicator expression, and distribution
Signal	Voltage	Voltage	Bulk fluorescence, can be defined by sensor	Fluorescence, can be defined by sensor	Fluorescence, can be defined by sensor

Because of these limitations of conventional MEAs, multi-electrode arrays fabricated on non-silicon substrates are highly desirable for combined electrical-optical measurements. New flexible arrays have been fabricated using microelectromechanical systems fabrication techniques^56^^,^^77^^,^^149^^,^^153^^–^^155^ on thin-film polymers including polyimide, parylene-C, and diverse silicone elastomers [Figs. 3(b)–3(d)]. Such arrays offer many advantages for combining electrophysiology and optophysiology.^67^^,^^156^^,^^157^ First, because of their mechanical flexibility, arrays can be arbitrarily positioned, and the connector can be moved out of the way to permit optical access near the array. Arrays can even be implanted vertically into the tissue, and the portion of the array outside the brain can be bent so the connector is placed to the side, permitting short-working-distance objectives to be positioned directly adjacent to the array. Second, the polymer substrates used to fabricate these arrays are not intrinsically light-sensitive, avoiding the trouble with light artifacts commonly experienced with silicon arrays. As mentioned above, this feature makes these arrays particularly appropriate for combination with multiphoton imaging or other imaging modalities that require pulsed excitation light. Third, a wide variety of partially or fully transparent flexible materials are used to fabricate these new MEAs, making them of especial interest for optical measurements. Many silicone-based polymers are optically transparent and thus permit optical access directly through the array. Likewise, polyimide and parylene are translucent, albeit they may exhibit some autofluorescence, depending on the excitation and emission wavelengths used for optical recording.^158^ Finally, transparent conductive materials, such as graphene or iridium-tin-oxide, provide the opportunity for fully transparent arrays, in which the electrode sites are themselves transparent.^150^

The advantages of flexible arrays are accompanied by some challenges. First, the density of contacts achievable on flexible MEAs is in general lower compared with similarly sized silicon arrays. This is because the size of features that can be reliably achieved when structuring conductive material on flexible substrates is in general larger than those achievable on silicon. Likewise, multiplexing has been increasingly used to increase the density of silicon arrays and can also be implemented on flexible MEAs.^159^ However, the addition of silicon-based transistors to flexible substrates renders them sensitive to light. The lack of multiplexing reduces the number of contacts that can be simultaneously recorded using a flexible array, mainly due to the size of the connector necessary to connect the probe to an amplifier for a non-multiplexed MEA. Superficially, this will lower the yield of recorded neurons for flexible as compared to silicon MEAs; however, the yield scales with the number of contacts and the development of high density flexible arrays has seen considerable progress in recent years.^64^^,^^77^^,^^157^^,^^160^^,^^161^ Practically, because the arrays are highly flexible, it can be a challenge to target them to the deep areas of interest in the brain, and special methods of insertion must be used depending on the desired site of implantation. It is also a challenge to chronically integrate flexible arrays with the rigid cranial bones, so the flexible array is not abraded during motion of the brain, and an array relative to the skull and connectors is held rigidly on the head. These are concerns for long-term chronic implants in larger animals with greater relative motion between brain and skull. Despite these challenges, the introduction of flexible and transparent electrode arrays has opened the door to many novel combined electrical and optical studies of brain function.

## 4 Diverse Possibilities for Combined Approaches

The diversity of recording techniques provides flexibility to determine which combinations are ideal for a specific experimental question. Of primary concern are the required spatial and temporal resolution, the signals of interest, the target brain areas, and the desired coverage. Recording electrically and optically from the same population of cells can provide complementary information about local neural circuit activity. On the other hand, recording from different brain locations opens the possibility to monitor the activity of multiple distributed populations and track the transformation and routing of signals across distributed brain networks. Given the range of electrical and optical recording approaches, there are many possible combinations. To simplify, we will focus on four main configurations that have been applied recently and illustrate the power of multimodal experiments to capture brain dynamics across scales. We will highlight recent state-of-the-art applications that address the contribution of single neurons, or classes of neurons, to local and distributed population dynamics in the healthy mouse brain and discuss how they inform and enhance our understanding of brain dynamics.

### 4.1 Integrating Electrophysiology and Photometry

**Fig. 4:**
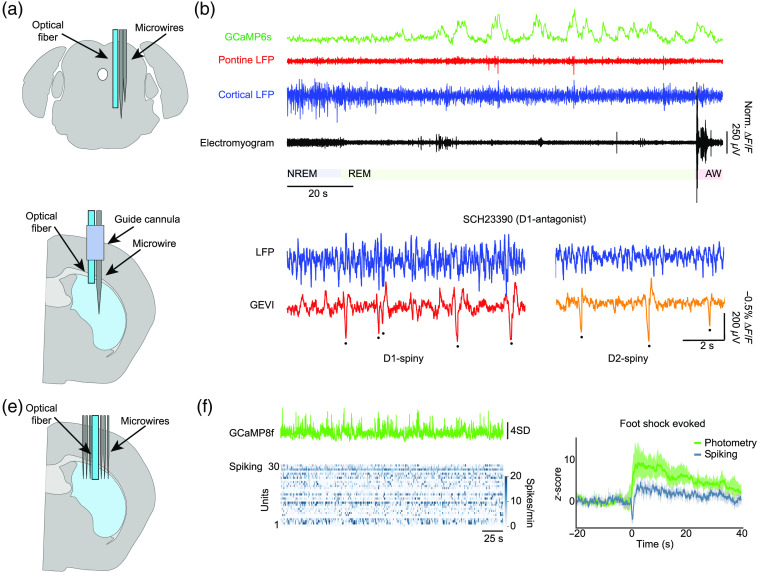
Combining MEAs with fiber photometry. (a) Two electrodes were coupled to a fiber-optic cannula and implanted in the mesopontine tegmentum to simultaneously measure the LFP from two distinct nuclei while optically monitoring calcium signals in cholinergic neurons. (b) Simultaneous recordings during the sleep-wake cycle in freely behaving mice indicated that bouts of activity in cholinergic neurons (GCaMP6s) co-occur with faster pontine waves (P-waves) during REM sleep. (c) A metal wire was coupled to a fiber-optic cannula and advanced through a guide-tube to simultaneously measure the LFP and optically monitor the bulk activity of specific populations of striatal MSNs. (d) Expression of GEVI in D1- or D2-receptor expressing MSNs permitted the specific contribution of these distinct classes to the LFP to be isolated and events obscured by the bulk nature of LFP were rendered visible (deflections in fluorescence traces marked by dots). The selective D1-antagonist SCH23390 was used to evaluate differential effects on D1- and D2-receptor expressing MSNs that are undetectable at the level of the LFP. (e) A bundle of microwires was formed around a fiber-optic cannula and implanted into the dorsomedial striatum. (f) Population calcium dynamics and the spiking activity of striatal neurons were simultaneously measured. Calcium signals in striatal neurons were found to mainly reflect non-somatic calcium signals, and spiking and calcium signals were differentially modulated by multiple behavioral events, such as a lever-press, an air puff to the animal’s face, or a foot shock (latter shown on the right). Data shown in (b), (d), and (f) are adapted with permission from Refs. 163, 164, and 165, respectively.

In the first combination, an optical fiber can be coupled to an electrode or MEA (often referred to as an “optrode”)^162^ to perform electrophysiology and fiber photometry in the same population [Fig. 4(a)]. Photometry is a popular optical method to record mesoscale activity, e.g., fluorescent indicator signals from a neuronal population, because of its relative simplicity and the fact that it enables optical recordings from deep regions of interest.^166^^–^^168^ Photometry can also be combined with fMRI.^132^^,^^143^ One promising integration of photometry with electrophysiology is co-implantation of fiber-optic cannulas with wire electrodes (Fig. 4). Whereas photometry measures bulk fluorescence signals and does not permit cellular-resolution imaging, selective expression of fluorescent proteins in a cell population of interest (based on genetic or anatomical considerations) makes it possible to relate the activity of a specific cell population to the bulk electrical LFP signals.

For example, Patel et al.^163^ constructed an optrode from a pair of microwire electrodes and a fiber-optic cannula [Fig. 4(a)]. They implanted the optrode in the mesopontine tegmentum, where GCaMP6s was expressed in cholinergic neurons. This combined approach enabled them to monitor fast electrical events and relate them to the activity of cholinergic neurons in freely moving mice across the sleep-wake cycle [Fig. 4(b)]. They found that cholinergic activity co-occurred with fast, electrical pontine waves (P-waves) during rapid eye movement (REM) sleep, a relationship that had been previously suggested based on electrophysiological and pharmacological studies^169^^,^^170^ but which could be directly demonstrated in their study using simultaneous opto- and electrophysiology. Using a similar approach, Marshall et al.^164^ advanced an optrode constructed from a single microwire electrode and a fiber-optic cannula through a guide-tube into the dorsal striatum of a mouse [Fig. 4(c)]. By expressing a GEVI in different populations of striatal medium spiny neurons (MSNs), it was possible to resolve cell-type specific contributions to local population activity that were obscured by the bulk nature of the LFP^164^ [Fig. 4(d)]. The cell-type specific expression of a GEVI into identified subpopulations of MSNs revealed distinct dynamics that were difficult to resolve at the level of the LFP. These two approaches highlight the power of combined photometry and electrophysiological recordings to better understand the cellular components of population events that are obscured when relying on a single modality. The refinement of such approaches will continue to reveal the cellular constituents of mesoscale LFP phenomena and may lead to the discovery of reliable signatures that can be applied to interpret EEG and LFP signals from human recordings where genetic and optical tools are not yet feasible. In a related, but different approach, Legaria et al.^165^ constructed a multi-electrode-fiber implant to monitor simultaneous calcium dynamics and spiking activity in a population of striatal neurons. They found that bulk photometry and neuronal spiking exhibited distinct response profiles to behavioral and sensory events, including lever-press, an air puff to the face, or a foot shock. Rather than simply reflecting APs in the local population, the photometry signal reflected non-somatic calcium dynamics. While this study motivates caution when simply equating photometric signals to neuronal APs, it also opens the door to investigating subcellular processes. For example, by targeting the expression of genetically encoded activity indicators to somas, dendrites, or axonal compartments, investigators could disentangle distinct subcellular contributions. Likewise, in combination with alternative indicators, such as sensors for specific neuromodulators, or cellular or transcriptional signaling molecules, such as ATP, cAMP, Arc, or c-Fos, experiments could be targeted to investigate subcellular processes correlated to distinct population events. Likewise, for many brain areas, it is still largely unknown how specific cell populations contribute to and are affected by local and long-range synchronization at different time scales. Therefore, optrode approaches can provide valuable data to better understand how the activity of a particular cell type (e.g., local cholinergic neurons or defined interneurons) or a specific projection path (e.g., the axons of a thalamocortical projection) is differentially engaged in faster events in the local population. Photometry of voltage indicators has also been used to assess fast, inter-areal synchrony in a cell-type specific manner,^171^ and similar approaches in combination with electrical recordings could disentangle the synchronization of specific subpopulations within the ongoing dynamics of the network. In addition, the development of high-density multi-fiber arrays enables simultaneous monitoring of local population signals in distributed circuits,^88^ and the possible combination of this approach with multi-electrode recordings could facilitate the bridging of cell-type-specific activity profiles with additional dynamics, such as the synchronization between populations on faster time scales.

### 4.2 Integrating Electrophysiology and Widefield Imaging

**Fig. 5:**
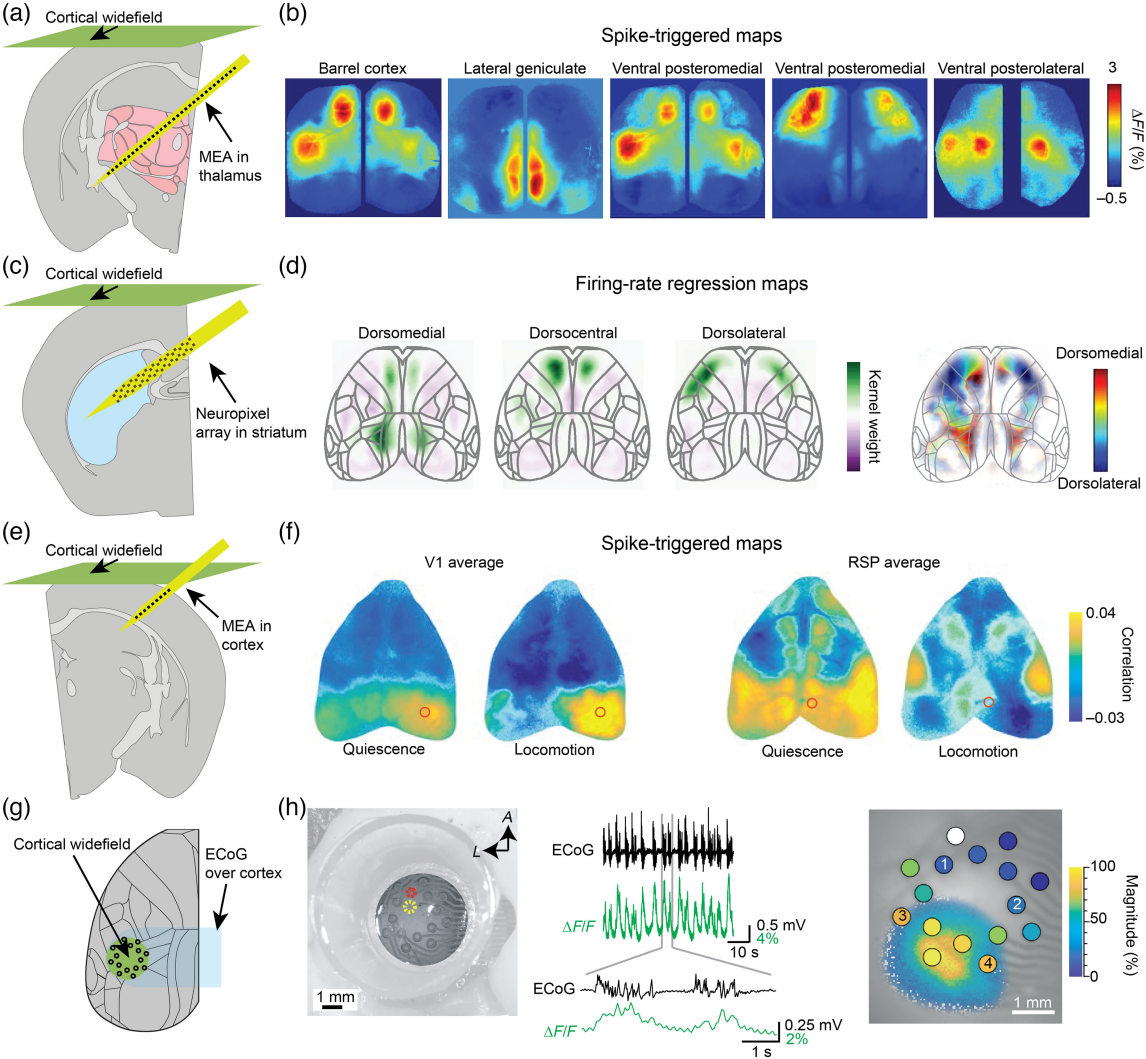
Combining MEAs and widefield imaging. (a) A linear MEA was implanted into the cortex and thalamus while simultaneous widefield imaging was performed to measure calcium dynamics in populations of cortical neurons. (b) The APs of isolated cortical and thalamic units were used to align the simultaneous cortical activity, revealing distinct patterns of widespread cortical activity for these individual neurons. (c) A Neuropixels array was implanted into the striatum while the widefield calcium signals of cortical neurons were monitored. (d) Individual striatal units revealed distinct topographic patterns of cortical activity depending on their medial to lateral position in the striatum, in agreement with expectations from anatomy. (e) Linear array recordings were performed in primary visual cortex (V1) or retrosplenial cortex (RSP) while widefield calcium signals were simultaneously monitored across the dorsal cortex. (f) Individual neurons within a given area were correlated with distinct patterns of distributed activity across the dorsal cortex. The coupling between individual neurons and the distributed cortical activity patterns was modulated by the behavioral state of the animal. (g) A transparent ECoG array was chronically implanted over somatosensory and visual areas of the mouse, permitting electrical and optical access to the underlying cortex. (h) Widefield calcium imaging of cortical neurons with simultaneous ECoG recordings enabled calcium signals in excitatory neurons to be monitored while faster LFP signals were recorded from the surface of the brain. The electrical and optical visual responses were in tight correspondence, showing the largest activity over posterior, visual regions. Data shown in (b), (d), (f), and (h) are adapted with permission from Refs. 148, 146, 145, and 67, respectively.

A second possibility combines electrical recordings using a penetrating MEA in a region of interest with simultaneous monitoring of large-scale activity across the dorsal cortex using widefield fluorescence imaging (Fig. 5). Mesoscopic widefield imaging permits monitoring of activity across large FOVs, and in the mouse, it can be performed even through the intact skull.^172^^,^^173^ This enables imaging of nearly all the dorsal cortex using contrast based on intrinsic signals related to hemodynamics or fluorescence signals in transgenic animals expressing indicators in populations of interest. Widefield imaging has also been performed with simultaneous fMRI, enabling whole-brain hemodynamic activity to be correlated to ongoing cortical activity.^144^ In addition, the possibility to target expression of the fluorescent indicator to a cell population of interest can be used to gain additional specificity while maintaining broad coverage. Several studies have used transgenic mice with GCaMP expression mainly in L2/3 but other mouse lines with distinct layer- or cell-type specific expression can be used in a similar manner, such as genetically specified long-range projection neurons or neurons of a specific inhibitory cell class.^174^^–^^176^ In combination with electrophysiology, this permits one to relate local activity from a cortical or subcortical area of interest to the ongoing activity of large portions of the dorsal cortex. Experiments using this approach can investigate how the activity of single cells, or small local populations, covaries with distinct spatiotemporal patterns of widespread cortical activity. Using commonly available rigid silicon probes, targets must be chosen so the array can enter the brain at an angle that still permits the objective lens to be positioned at an appropriate distance from the cortical surface.

For example, linear silicon arrays have been implanted at an angle permitting imaging, while passing through cortex and thalamus^148^ [Fig. 5(a)]. The APs of units recorded at different positions on the array can be used to estimate the pattern of cortical activity they correlate with, producing spike-triggered activity maps, or “cortical fingerprints” [Fig. 5(b)]. These fingerprints vary based on the location of the electrically recorded neuron and presumably relate to the large-scale whole-brain network states, in which the neuron participates. Likewise, a Neuropixels probe^36^ can be inserted to traverse the dorsal striatum from medial to lateral [Fig. 5(c)], revealing a highly ordered topography of cortical activations and suppressions coinciding with the activity of striatal cells of a given anatomical position [Fig. 5(d)].^146^ The variation in cortical fingerprints for different striatal positions matches the anatomical organization of cortical projections to the striatum and enables the assessment of large-scale functional anatomy.^177^ Another important question is how these fingerprints vary dynamically as a function of the state of the animal. One study investigated how the cortical fingerprints of single neurons in primary visual cortex (V1) and retrosplenial cortex (RSP) vary depending on whether the animal was actively moving or passively resting [Fig. 5(e)].^145^ Not only were the cortical fingerprints of single neurons in these areas idiosyncratic but they also altered their coupling to the rest of the brain when the animal transitioned from quiescence to a state of active locomotion [Fig. 5(f)]. Recent work has also used MEA recordings of the hippocampus in combination with cortical widefield imaging to investigate hippocampal–cortical interactions during sharp-wave ripples.^156^^,^^178^^,^^179^ These studies enrich our perspective on the activity of single neurons, not only studying their dynamics in isolation but also revealing how the activity of single neurons is coordinated with large-scale patterns of activity distributed across the cortex.

Widefield imaging can also be performed through chronically implanted transparent ECoG arrays^67^ [Fig. 5(g)]. Local dynamics on a fast time scale can be related to cortex-wide activity in a cell-type specific manner [Fig. 5(h)]. For example, to better understand the origins of macroscopic electrophysiological phenomena, one can estimate how synchronization at different time scales corresponds to the activity of a specific class of neurons [Fig. 5(h)]. While widefield imaging is technically relatively simple, employing single-photon excitation and fluorescence imaging with a camera, care must be taken to evaluate and correct for the potential artifacts introduced by intrinsic, especially hemodynamic, signals,^172^^,^^180^^,^^181^ as well as in identifying the neuronal source of the recorded signal.^182^ Most studies relating local cellular activity to the activity of distributed networks have focused on electrophysiological recording from a single subcortical source due to constraints in simultaneously performing optical imaging;^145^^,^^146^^,^^148^^,^^156^ however, advances in flexible MEAs and new surgical preparations will enable simultaneous optical investigation in combination with multi-site electrophysiology.

### 4.3 Integrating Electrophysiology with Cellular Imaging

On a finer level, one can combine electrophysiology with MEAs with cellular imaging, using, for example, two-photon microscopy or portable miniscopes. Two-photon imaging is the current gold-standard for cellular or subcellular *in vivo* imaging, providing sub-micrometer resolution and allowing measurement of fluorescent indicators up to 1 mm deep in brain tissue.^108^^,^^183^ The development of a large range of indicators and technical advancements of microscopes have made it possible to record neural activity across scales from axonal boutons and dendritic spines to large populations of more than hundred-thousand neurons (reviewed in Ref. 28).

**Fig. 6:**
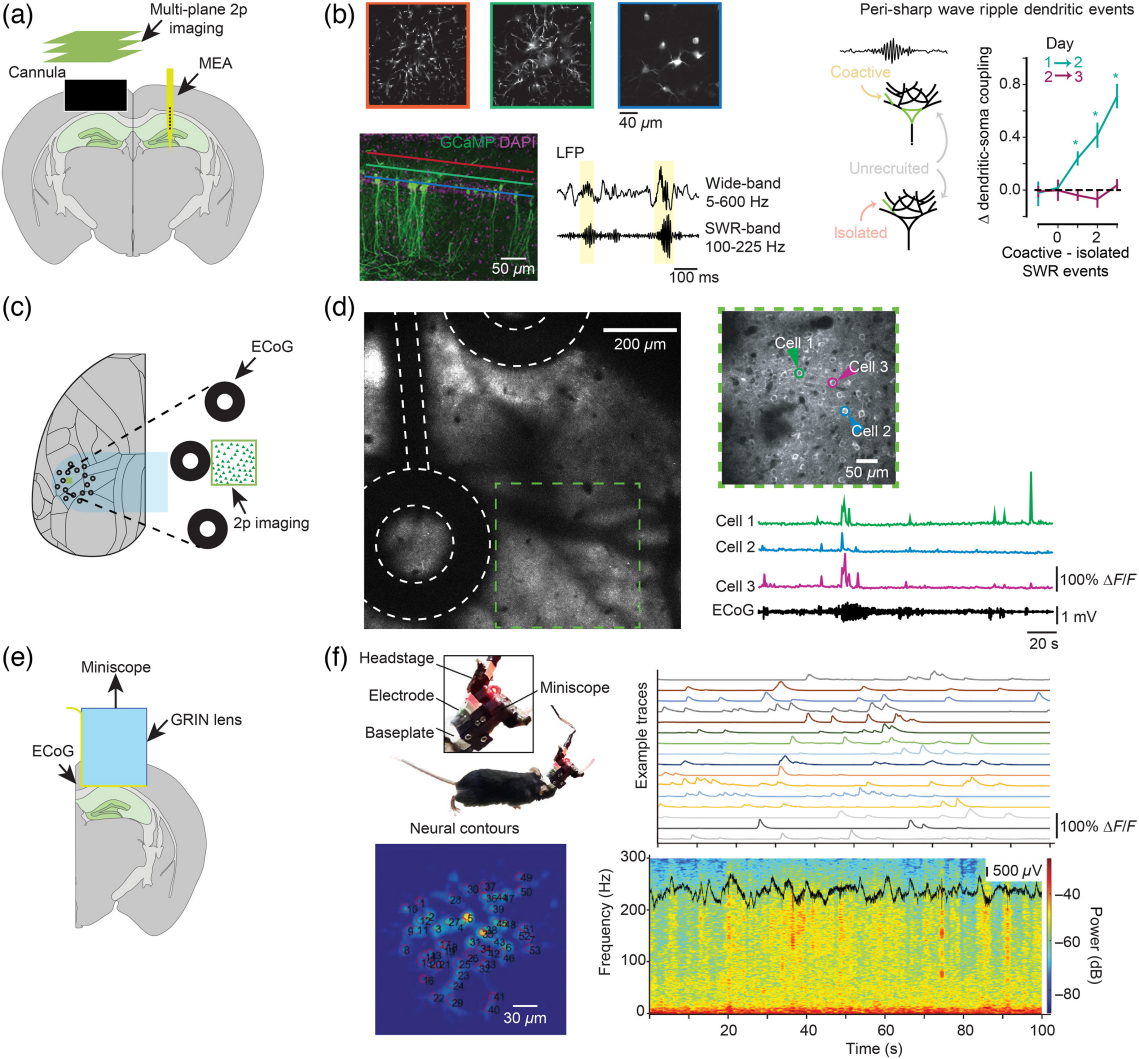
Combining MEAs and cellular imaging. (a) Linear array recordings in the dorsal hippocampus were performed simultaneously with two-photon imaging of calcium transients in pyramidal cells in the CA1 layer of the contralateral dorsal hippocampus. Linear arrays were used to detect fast synchronization (ripple events, on the order of tens of ms) to the dendritic calcium events of CA1 pyramidal cells. (b) Dendritic coupling in CA1 pyramidal cells estimated with multiplane two-photon imaging correlated with the number of global dendritic calcium events that occurred during ripples 24 h prior. (c) A transparent ECoG array was used to record electrical activity from the surface of the brain, while simultaneously monitoring cellular activity using two-photon calcium imaging from cortical pyramidal neurons. (d) The activity of single neurons could be related to the ongoing pattern of electrical synchronization on faster times scales. (e) An MEA was integrated into a miniscope, permitting simultaneous electrical and optical recording from CA1 populations in freely moving mice. (f) Population synchronization was assessed with LFP recordings while single neuron activity was monitored with a genetically encoded calcium indicator. Data shown in (b), (d), and (f) are adapted with permission from Refs. 147, 67, and 185, respectively.

Optical recordings of single-cell calcium dynamics can, for example, be related to known electrophysiological markers of population activity, such as sharp-wave ripples (SWRs) in the hippocampus.^184^ Rolotti et al.^147^ recorded calcium transients in dendrites and somata of hippocampal neurons together with the LFP in the contra-lateral hippocampus [Fig. 6(a)]. They found that during learning, the co-activity of dendritic branches and soma during SWRs was predictive of dendrite-soma coupling on the next day [Fig. 6(b)]. In epilepsy research, combining LFP recordings with two-photon imaging can monitor the spread of seizures for different cell types and cortical layers^186^^,^^187^ and detect activity abnormalities before the detection of the seizure in the LFP.^188^^,^^189^ Aside from these examples, there are many other prominent LFP features whose cellular and subcellular correlates are only partially known. Patterns of cortical synchronization in the beta and gamma frequency ranges, signatures of sleep stages, such as slow waves, spindles, and prominent thalamic activity patterns, such as bursting and tonic firing, are all population phenomena for which their cellular and subcellular effects in distinct structures are only vaguely understood.^5^^,^^6^^,^^11^^,^^190^^–^^197^ Recording of LFP or ECoG signals in combination with cellular imaging could reveal the cellular activity generating, recruited by, or participating in these distinct mesoscopic phenomena. Better understanding of these population dynamics, which are of high clinical relevance,^198^^,^^199^ promises to improve both clinical biomarkers, as well as bridge our cellular understanding of neuronal dynamics in model species with the mesoscopic signals measurable in the human brain.

Typically, the combination of two-photon imaging with electrical recordings is challenging because of the difficult geometric arrangement of objectives with short working distance and rigid electrodes, as well as the light-induced electric artifact.^200^ Therefore, many studies performed acute experiments with glass pipettes or recorded electrical activity spatially or temporally separated from the optical recordings.^53^^,^^147^ The development of flexible recording electrodes (reviewed in Refs. 33 and 201) is now enabling more flexible electrode placements and artifact-free combinations of two-photon imaging and electrical recordings. For example, flexible transparent ECoG arrays allow optical recording of neurons below the recording array to investigate the relationship between the activity of single, identified neurons and macroscopic electrical signals [Figs. 6(c) and 6(d)].^67^^,^^202^^,^^203^ With flexible linear arrays, it is also possible to insert the array vertically into the tissue below the cranial window.^204^ This approach has been used, for example, to monitor the tissue damage caused by array insertion.^157^ However, such an approach can enable dense optical recording of cellular and subcellular activity (such as local dendritic events or axonal events arising from a known projection) in combination with electrical monitoring of local and long-range synchronization, including from subcortical sources.

These advancements of flexible electrodes are paralleled by improvements of two-photon microscopes to record from large populations of neurons across multiple areas^205^^,^^206^ and in various locations of the brain.^207^ By combining these advanced optical and electrical recording modalities, new insights into the relationship of single neurons with the ongoing population activity across scales can be gained. For example, longitudinal data could be acquired to monitor how behavioral state, experience, or learning alter the relationship between optically monitored cellular or subcellular features, such as spines, dendrites, or axons, and population events, such as local or long-distance synchronization at specific temporal scales. In addition, the array can be placed in a distant site, for example, the thalamus, while the cortex is densely imaged, permitting cellular and population activity in the thalamus to be related to population, cellular and subcellular cortical activity. Such a combination could be used track the flow of information across a sensory hierarchy, for example, by recording electrical activity in the visual thalamus or superior colliculus, while simultaneously monitoring cortical activity in multiple visual processing centers using a multi-area two-photon microscope.^116^^,^^119^ The possibility to define the expression of an indicator in a specific population of interest further increases the specificity of the questions that can be addressed with this technique.

### 4.4 Integrating Electrophysiology and Miniscopes

Lastly, it is possible to position a planar MEA below the GRIN lens of a head-mounted miniature widefield microscope to record population synchrony in addition to dense, unbiased imaging of cellular activity [Fig. 6(e)]. Like the combination of electrophysiology and two-photon imaging, this approach provides spatially resolved recordings of fast electrical activity (LFP and MUA) together with the slower measurement of activity of all the cells lying in the focal plane of the miniscope. Imaging can be performed broadly, to assess a large, unbiased population of cells, or can be targeted to a specific population to relate their activity to the spatially distributed electrical activity. Most compellingly, such a combination enables recordings in freely moving subjects, enabling detailed neural measurements during unconstrained, naturalistic behaviors. In the example in Figs. 6(e) and 6(f), the integration of a flexible MEA with a miniscope enabled the investigation of densely sampled pyramidal neurons in the CA1 subregion of the dorsal hippocampus in combination with simultaneous measurement of population synchrony assessed with the LFPs recorded on the MEA while the animal was freely moving [Fig. 6(f)].^185^ Such an approach could answer questions about the fraction of a local population engaged during synchronization, about which cells are engaged when oscillations of different frequency occur, or about how cell types defined genetically or by projection patterns differentially engage with population activity. Improvements in head-mounted multiphoton microscopes also provide the basis for future multimodal investigations using these devices.^208^^–^^210^ In general, there is a high degree of flexibility for combining electrical with optical recordings from the brain, not only in terms of the electrical and optical measurements made but also in terms of the areas of interest and the signals that are optically monitored.

## 5 Linking Scales Together

We have illustrated the power of combined electrical and optical recordings for the study of brain function and highlighted recent work demonstrating its potential. We believe that it is a particularly fruitful time to perform multimodal and multiscale studies because of the remarkable recent advances in electrode and imaging technologies, as well as molecular probes. We look forward to the new perspectives that future studies will provide on large-scale brain dynamics and the integration of cellular signals into whole-brain activity patterns in the awake, behaving animal (see Box 1).

**Fig. 7:**
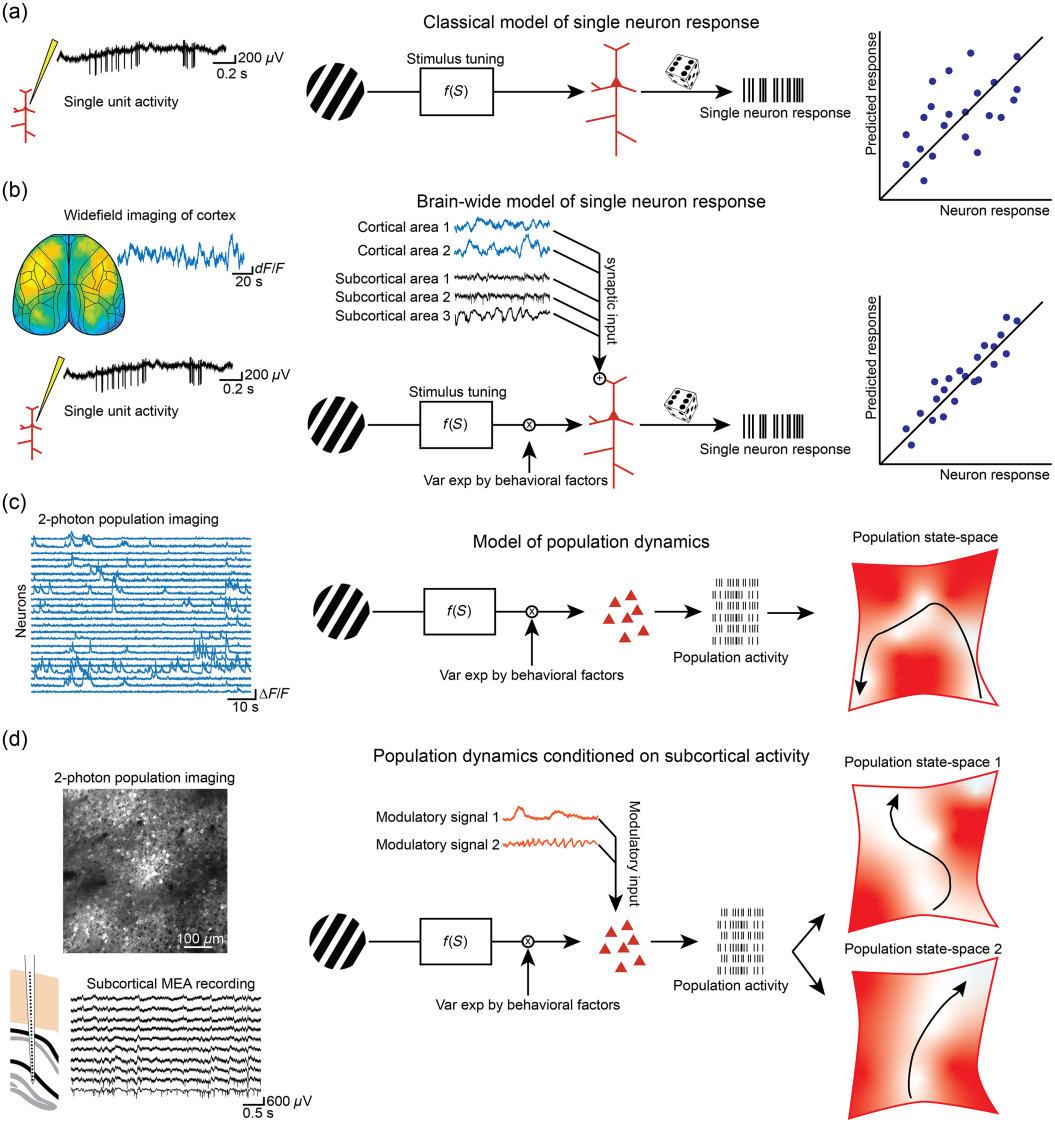
Integrating information across scales. (a) Classical analysis in systems neuroscience has attempted to understand the activity of single cells as a function of the sensory input delivered to the subject (f(S)). (b) Multimodal studies that combine electrical and optical measurements to provide access to distributed and diverse brain signals can permit multi-factorial models of single-neuron activity that are based not only on the sensory stimulus but also on the additional signals from other brain areas, specific cellular populations, or modulatory input. (c) Moving beyond single cells, population recordings have enabled analysis of the joint dynamics of neuronal groups. (d) Multimodal recordings of populations in conjunction with measurements of the activity of long-range modulatory or synaptic inputs can permit the joint activity of neuronal groups to be determined as a combination of both external and internal factors that contribute to the evolution of the population dynamics.

Neuroscience has classically treated neurons as individual units of computation in the brain and sought to understand the brain through the isolated activity of single neurons, local populations, or brain areas. By recording the activity of single neurons while manipulating sensory, motor, or cognitive variables, it has been possible to characterize tuning properties or transfer functions that relate these external variables to the activity of single cells [Fig. 7(a)]. This approach has taught us a great deal about what external factors neurons respond to, however, the large degree of variance of neuronal responses in such models has traditionally been treated as noise. We now have extensive anatomical and physiological evidence that the variance in single neuron responses is highly structured and that the brain is continually active, generating intrinsic activity patterns that interact with externally arising events in a complex and state-dependent way.^122^^,^^123^^,^^211^ New techniques and approaches for multi-site and multimodal investigation of brain activity have enhanced our perspective on integrated brain function, helping us to understand not only the diverse physiological processes underlying brain activity but also how the activity of individual neurons and neuronal populations are coordinated with brain-wide activity patterns. The rich heterogeneity of neurons across the brain, and within a given local circuit, are increasingly appreciated, and new high-throughput methods to characterize the diversity and specificity of neurons and the networks in which they participate will continue to reveal the brain’s intricate functional and anatomical organization.^8^^,^^9^^,^^14^^,^^212^^–^^214^ These findings and new technologies have led to an increase in population recording and contributed to a shift away from single neuron models and toward understanding neuronal activity in terms of the joint dynamics of the population in which they are embedded. We believe that multimodal studies can augment such models by contributing important additional explanatory variables for single neuron and population activity. In the first case, rather than modeling the activity of single cells based solely on experimenter-controlled variables, we can use additionally monitored intrinsic signals, such as the activity of distant brain sites, neuromodulatory tone, or the activity of non-synaptic partners, such as astrocytes [Fig. 7(b)]. In the extreme, such a model could be used to condition not only the activity of a single neuron but also the dynamics of the population in their joint activity space [Fig. 7(c)]. For example, a modulatory input could dramatically shift the population dynamics from one dynamic state space to another with radically different consequences for local circuit transformations and the information signaled to downstream partners [Fig. 7(d)]. In the end, such a model might be able to help explain the dynamic transformation and routing of sensory signals in coordination with the current state and objectives of the experimental subject, to better understand the variability of behavioral dynamics on a moment-to-moment basis.

While technical advances are making multimodal studies more practical, the data collected from multimodal studies also pose considerable hurdles in their analysis. Most analysis tools are focused on signals of a single spatial or temporal resolution, and, to date, most multiscale studies have been correlational. The analysis approach commonly employed by multimodal studies has been to either look at time-locked averages around events detected with one modality or to use a signal from one level of organization to predict a different signal at a higher or lower level of organization.^125^^,^^128^^,^^136^^,^^165^ In general, these analyses do not have a model of how the two signals are causally related or how the signals or events themselves are generated. Such an approach can reveal many aspects of integration and functional coupling in brain networks. However, ultimately it is desirable to know how activity interacts across the levels of brain organization, and how signals, events, and even large-scale activity patterns are generated and dynamically evolve and transition.^215^ For example, the evidence for behavioral and experience-based modification of coupling across levels indicates a complex inter-relationship that is dynamic and is likely to depend on a variety of inter-related factors, such as the animal’s motivation, goals, recent history, and previous experience.^145^^,^^147^ Linking brain activity across scales in such a way requires new analytic and modeling approaches that facilitate causal interactions across levels of brain organization, as well as a better understanding of the phenomenological aspects of animal’s behavioral patterns, the dynamics of behavior, and of the behavior-associated brain activity.^216^^,^^217^ Ultimately, it will be necessary to augment multimodal investigations with perturbations at identified scales to understand how such perturbations propagate across levels or lead to dynamic switching of large-scale brain states. While tools exist to manipulate brain activity at different scales, the degree to which such manipulations give rise to physiological meaningful activity patterns is only partially understood. While targeted perturbations of single cells,^218^^,^^219^ or sequences of physiologically characterized cells appear to approach naturalistic manipulations, manipulations at larger scales are less well understood. An interesting question is to which degree it is possible to manipulate macroscale, whole-brain activity patterns in a meaningful way to the animal. For example, can large-scale activity patterns be biased or reproduced *de novo* to evaluate their effects on isolated cells or populations? Given the varied specific and pronounced behaviors that can be evoked when stimulating small groups of cells, for example, in the hypothalamus,^220^ amygdala,^221^ or periaqueductal gray matter,^222^^–^^224^ it is conceivable that large-scale changes in brain-state are linked to the slow variation in activity in some circuit or arise in the competition between antagonistic populations of cells each vying to drive the brain’s large-scale dynamics. Neuromodulatory systems are likely to play a key role in the orchestration of such dramatic shifts in brain-wide activity, given their extensive projections and diffuse signaling. It is likely that such changes are driven at distinct time scales—from the moment-to-moment variations in vigilance and motivation, to short-term and long-term goals, circadian rhythms, and the more temporally extended time course of the individual’s life. We believe that the combined use of optical and electrical measurements will continue to reveal new details and provide unique perspectives on integrated brain function.
